# Two Cases of Meningococcal Disease in One Family Separated by an Extended Period — Colorado, 2015–2016

**DOI:** 10.15585/mmwr.mm6712a4

**Published:** 2018-03-30

**Authors:** Emily Spence Davizon, Heidi M. Soeters, Lisa Miller, Meghan Barnes

**Affiliations:** ^1^Colorado Department of Public Health and Environment, Denver, Colorado; ^2^Division of Bacterial Diseases, National Center for Immunization and Respiratory Diseases, CDC.

On April 26, 2015, a case of meningococcal disease in a woman aged 75 years was reported to the Colorado Department of Public Health and Environment (CDPHE). As part of routine public health investigation and control activities, all seven family contacts of the patient were advised to receive appropriate postexposure prophylaxis (PEP) to eradicate nasopharyngeal carriage of meningococci and prevent secondary disease (*1*), although it is not known whether the family contacts complied with PEP recommendations. Fifteen months later, on June 6, 2016, CDPHE was notified that the grandchild of the first patient, a male infant aged 3 months who lived with the first patient, also had meningococcal disease. The infant’s immediate family members (parents and one sibling) were among family contacts for whom PEP was recommended in 2015. *Neisseria meningitidis* isolates from both patients were found to be serogroup C at the CDPHE laboratory. Whole genome sequence (WGS) analysis at CDC found that both isolates had the same sequence type, indicating close genetic relatedness. These cases represent a possible instance of meningococcal disease transmission within a family, despite appropriate PEP recommendations and with a long interval between cases.

## Investigation and Results

On April 24, 2015, the first patient was evaluated at hospital A for aphasia, rigors, chills, and fever. She was hospitalized and treated empirically with ceftriaxone and azithromycin. Blood specimens collected before antibiotic initiation were culture-positive for *N.* *meningitidis*; Gram stain of the patient’s cerebrospinal fluid (CSF), collected after initiation of antibiotics, revealed gram-negative diplococci, characteristic of *Neisseria* species; however, bacterial cultures showed no growth after 7 days. CDPHE was notified of the case on April 26, 2015. Once *N. meningitidis* was identified, the patient received 10 days of ceftriaxone therapy. She was discharged to a skilled nursing facility after 7 days and recovered. The blood isolate was determined to be serogroup C via slide agglutination and real-time polymerase chain reaction (real-time PCR) testing at the CDPHE laboratory.

Fifteen months later, on June 4, 2016, the grandson of the first patient, aged 3 months, was evaluated in the emergency department of hospital B for fever and decreased alertness. A blood specimen was obtained, empirical ceftriaxone was administered, and the patient was airlifted to hospital C, where petechiae were noted, and a lumbar puncture was performed. Gram stain of the patient’s blood revealed gram-negative diplococci, and blood culture was positive for *N. meningitidis*. No organisms were detected in the infant’s CSF. An assessment of the infant for complement component deficiency, which can increase risk for meningococcal disease (*2*), did not reveal any abnormalities. CDPHE was notified of the case on June 6, 2016. The infant recovered after 7 days of treatment with ampicillin. CDPHE laboratory determined the blood isolate from the infant was also serogroup C *N. meningitidis* via slide agglutination and real-time PCR.

Following the identification of the second patient, isolates from both the grandmother and the grandchild were submitted for WGS analysis at CDC. The two isolates had the same sequence type (sequence type 2006, clonal complex 103) and were more closely related to each other than to other isolates from sporadic cases within the same clonal complex. Antimicrobial susceptibility testing indicated both isolates were pansusceptible.

## Public Health Response

In 2015, after the report of the first case, a public health investigation was conducted by Jefferson County Public Health and CDPHE. Seven family contacts of the patient were identified and advised to receive PEP, consisting of oral ciprofloxacin for the six adult contacts and intramuscular ceftriaxone for one child contact.

In 2016, during the public health investigation of the second case, it was learned that the grandmother lived with her grandson and was his child care provider. Oral ciprofloxacin PEP was recommended for five adult contacts and oral rifampin for one child contact (five household contacts and one community contact). All five household contacts had previously been advised to receive PEP following the first patient’s illness in 2015. 

## Discussion

Meningococcal disease is a rare and serious illness; an average of 10 cases per year were reported in Colorado during 2011–2016. *N. meningitidis* is transmitted through direct contact with large-droplet respiratory tract secretions from persons with meningococcal disease or asymptomatic nasopharyngeal carriage (*2*).

Although the first patient lived with and cared for the second patient during the day, she was appropriately treated for meningococcal disease, and her family contacts, including the parents and sibling of the second patient, were appropriately advised to receive PEP; however, compliance with PEP recommendations was not known. These cases represent a possible instance of meningococcal disease transmission within a family, despite appropriate PEP recommendations and with an interval of 15 months between cases, with the second case occurring in an infant who was not yet born at the time the first case occurred. There have been some documented examples of household transmission of meningococcal disease (*3*–*7*); a review estimated the attack rate among household contacts who received appropriate PEP to be 1.1 cases per 1,000 household contacts (*4*).

The mechanism behind this instance of household transmission is unclear. An unidentified close contact of the grandmother could have been a close contact of the infant patient, or this strain of *N. meningitidis* could have been circulating asymptomatically in the wider community for an extended period. A third possibility is that PEP failed to eradicate carriage within the family, either because of incomplete compliance, nonsimultaneous administration, or incomplete eradication of carriage. Although estimates vary, 5%–10% of adults are colonized with *N. meningitidis* at any given time, and most colonized persons carry nonpathogenic strains and do not develop disease (*1*). Both disease treatment and PEP would be expected to eradicate meningococcal carriage (*1*,*8*). Although PEP was recommended and prescribed for all identified close contacts of the first patient, because compliance was not ascertained, it is not known whether all contacts received PEP. Because *N. meningitidis* can only survive on surfaces for ≤10 days (*9*), it is unlikely that environmental persistence of the bacteria contributed to transmission.

Household links between cases are not routinely documented as part of national meningococcal disease surveillance, and the frequency with which household transmission of meningococcal disease occurs in the United States is not easily known. The 15-month interval between these two cases is longer than in previous reports of household transmission (range = <1 day to 39 weeks) (*3*–*7*). However, some of these studies (*6*,*7*) limited their definition of secondary cases to a specific window of time after the first case, limiting these comparisons. WGS analysis confirmed the same sequence type in both cases, whereas older reports of multiple cases of meningococcal disease within households could determine that isolates were of the same serogroup but lacked the ability to determine the sequence type.

Vaccination of close contacts of patients with sporadic meningococcal disease in addition to PEP is not currently recommended in the United States for the prevention of secondary cases. Additional evaluations to estimate the secondary attack rate within households and efforts to improve documentation of PEP compliance would be helpful to assess existing recommendations for public health response to meningococcal disease cases in the current U.S. epidemiologic context.

**Summary**

**What is already known about this topic?**

Meningococcal disease is a rare and serious illness; an average of 10 cases per year were reported in Colorado during 2011–2016. Both disease treatment and postexposure prophylaxis (PEP) of close contacts of persons with meningococcal disease are expected to eradicate meningococcal carriage.

**What is added by this report?**

This report describes a possible instance of meningococcal disease transmission within a family, despite appropriate PEP recommendations (but without documentation of compliance), with a 15-month interval between cases and with the second case in an infant who was not yet born at the time the first case occurred. Whole genome sequencing was used to confirm the same sequence type in both cases, whereas older reports of multiple cases of meningococcal disease within households were often limited to the same serogroup, without ability to confirm the exact strain.

**What are the implications for public health practice?**

Vaccination of close contacts of sporadic meningococcal disease cases in addition to PEP is not currently recommended in the United States for the prevention of secondary cases. Additional evaluations to estimate the secondary attack rate within households and efforts to improve documentation of PEP compliance would be helpful to assess existing recommendations for public health response to meningococcal disease cases in the current U.S. epidemiologic context.
